# 小细胞肺癌免疫治疗研究进展

**DOI:** 10.3779/j.issn.1009-3419.2018.12.10

**Published:** 2018-12-20

**Authors:** 涛 于, 殿胜 钟

**Affiliations:** 300052 天津，天津医科大学总医院肿瘤科 Department of Medical Oncology, Tianjin Medical University General Hospital, Tianjin 300052, China

**Keywords:** 肺肿瘤, 免疫治疗, 临床研究, Lung neoplasms, Immunotherapy, Clinical development

## Abstract

小细胞肺癌（small cell lung cancer, SCLC）约占肺癌的15%，恶性程度高，侵袭性强，尽管其对放化疗比较敏感，但极易发生耐药，复发率高，后期治疗效果欠佳。近年来，免疫抑制剂展现了良好的抗肿瘤活性，特别是程序性死亡受体-1/配体-L1（programmed death receptor-1/ligand-L1, PD-1/L1）和细胞毒性T淋巴细胞相关抗原-4（cytotoxic T-lymphocyte-associated antigen 4, CTLA-4）检查点抑制剂的问世改变了肿瘤治疗的格局，同时SCLC具有高免疫源性，高突变负荷等免疫利好因素，免疫检查点抑制剂有望成为该领域治疗的重要突破口，本文将对SCLC免疫治疗的临床研究进展做简要综述。

## Abstract (en)

Small cell lung cancer (SCLC), which accounts for about 15% of lung cancer cases, is an aggressive disease characterized by rapid growth and early widespread metastasis. Despite sensitive to chemotherapy and radiotherapy, SCLC is vulnerable to get resistant and has high recurrence rates. In recent years, immunotherapy has shown good antitumor activity, especially programmed death receptor-1/ligand-L1 (PD-1/L1) and cytotoxic T-lymphocyte-associated antigen-4 (CTLA-4) Checkpoint inhibitors have changed the pattern of tumor treatment, and SCLC has high immunogenicity, high mutation load and other favorable immune factors, so immuno-checkpoint inhibitors may become an important breakthrough in SCLC treatment. This article will briefly review the clinical research of immunotherapy for small cell lung cancer.

小细胞肺癌（small cell lung cancer, SCLC）约占所有新发肺癌的15%，其中70%的SCLC患者在疾病确诊时已处于广泛期^[1]^，SCLC侵袭性极强，其肿瘤细胞倍增时间短，生长指数高，易合并副肿瘤综合征，复发转移几率高，广泛期患者的中位总生存期只有8个月-13个月，2年生存率不到5%^[2]^。近年来，免疫治疗在肿瘤治疗领域连创佳绩，相继写入多个癌种的指南推荐，本文将对SCLC免疫治疗的机理和临床研究进展进行综述。

## 1 SCLC免疫治疗机理

### 1.1 SCLC具有高突变负荷

研究^[3]^证实SCLC的发生与烟草暴露明显相关；而长期烟草接触可以导致肿瘤突变负荷（tumor mutation burden, TMB）升高^[4]^；另一方面，SCLC患者普遍存在癌基因的异常活化和抑癌基因的失活突变，特别是*TP53*和*RB1*的高频失活突变^[5]^，这两个基因均参与细胞DNA的修复，因此，它们的缺失可以导致基因组的高度不稳定性，同样造成肿瘤突变负荷增加。

### 1.2 SCLC具有高免疫源性

SCLC患者较易合并副肿瘤综合征（paraneoplastic neurological syndrome, NPS），近年来研究证实这种现象和自身免疫相关^[6]^，由于肿瘤细胞作为始动抗原诱发机体产生高度特异性Hu抗体，参与机体自身免疫反应，从而出现一系列的远隔效应，这种与原发病灶无关的神经内分泌现象往往提示这些患者具有激活的免疫系统。在一项纳入170例SCLC患者的多因素研究^[7]^中指出Hu抗体的存在是免疫治疗完全应答的独立预后因素，与没有合并NPS的患者相比，存在NPS的患者体内有高水平的Hu抗体，并且其主要组织相容性复合体（major histocompatibility complex, MHC）Ⅰ类蛋白没有下调，使得这部分患者对免疫检查点抑制剂更为敏感；另一方面，这种潜在的反应增强也可能导致免疫治疗相关的不良反应增加，严重的脑炎和重症肌无力的发生率明显升高。

### 1.3 SCLC具有低肿瘤淋巴细胞浸润

正常情况下，机体通过主要组织相容性复合体（major histocompatibility complex-Ⅰ, MHC-Ⅰ）将肿瘤细胞表面抗原呈递给免疫系统，因此MHC-Ⅰ类分子表达水平与细胞毒性T细胞（cytotoxic T-lymphocyte, CTL）抗原呈递明显相关，有研究^[8]^表明SCLC患者体内肿瘤细胞MHC-Ⅰ类表达减少或缺失，肿瘤淋巴细胞浸润（tumour-infiltrating lymphocytes, TILs）水平较低，并且大部分淋巴细胞分布在肿瘤边缘而不是肿瘤内部，即MCH-Ⅰ类抗原的低表达导致T细胞募集减少，从而造成SCLC患者肿瘤内TIL浸润减低，是免疫治疗应答的负性因素。

综上所述，SCLC患者肿瘤突变负荷普遍较高，同时部分患者合并副肿瘤综合，具有高免疫源性，此为免疫治疗的利好因素，而另一方面，SCLC患者体内肿瘤性淋巴细胞浸润比例较低，这又在一定程度上限制了免疫检查点抑制剂的疗效。

## 2 SCLC免疫治疗临床研究进展

### 2.1 一线治疗

作为最早获批的免疫检查点抑制剂Ipilimumab，首先在SCLC领域尝试一线治疗，这一全人源化的抗CTLA-4单克隆IgG1抗体，通过阻断T细胞表面CTLA-4与抗原提呈细胞上的配体CD80/CD86结合从而增强抗肿瘤免疫反应，CA184-041是由德国Martin Reck教授牵头开展的多中心Ⅱ期临床试验^[9]^，比较Ipilimumab联合紫杉醇/卡铂一线治疗晚期SCLC患者的疗效，其主要研究终点免疫相关无进展生存（immune-related PFS, irPFS）较单纯化疗显著提高；随后，该团队进一步开展了Ⅲ期研究CA184-156，纳入1, 132例晚期SCLC患者，对比Ipilimumab联合依托泊苷/顺铂（Etoposide/Cisplatin, EP）治疗广泛期SCLC的疗效^[10]^，与单纯化疗组相比，免疫联合组中位无进展生存期（median progression free survival, mPFS）及中位总生存期（median overall survival, mOS）均无获益，研究团队认为Ipilimumab只是在免疫活化阶段发挥作用，缺乏后续的免疫效应因子，不能有效激起足够的抗肿瘤免疫反应，是CTLA-4抑制剂一线治疗失败的主要原因。

Nivolumab是人源化抗PD-1单克隆IgG4单抗，通过结合T细胞表面的PD-1受体阻止抗肿瘤免疫反应，作为免疫效应阶段的药物与Ipilimumab单抗联合应用可以增强免疫治疗的效果，但在SCLC一线治疗领域还没有两者联合的研究数据报道；NCT03382561是Nivolumab单药联合标准化疗的Ⅱ期随机研究，计划入组150例患者随机分配到EP标准治疗组，或者Nivolumab联合治疗组，该研究将于2018年完成患者招募，初步结果预计将于2020年公布。

Pembrolizumab是另一种人源化PD-1单克隆IgG4抗体，在SCLC一线治疗中开展了多项研究：Ⅰ期多队列研究KEYNOTE011中的队列E评估Pembrolizumab联合标准化疗方案一线治疗SCLC的疗效，初步结果预计在2020年公布；REACTION（NCT02580994）是一项在未经治疗的广泛期SCLC患者中开展的多中心、开放、随机对照、Ⅱ期临床试验，旨在比较依托泊苷+顺铂/卡铂联合或不联合Pembrolizumab的疗效差异，主要研究终点为PFS，初步结果也将在2020年公布；另外一项Ⅲ期随机、双盲、安慰剂对照临床实验KEYNOTE-604也是对比Pembrolizumab联合标准化疗方案一线治疗广泛期SCLC的研究，主要研究终点是PFS和OS，初步结果将在2019年公布。

Atezolizumab是抗PD-L1的人源化单克隆IgG1抗体，2018年世界肺癌大会（World Conference on Lung Cancer, WCLC）公布其Ⅲ期双盲、随机对照IMpower133研究数据^[11]^，入组的403例初治广泛期SCLC患者，1:1随机接受Atezolizumab联合EP四个周期诱导化疗后序贯Atezolizumab维持治疗，或者安慰剂联合EP序贯安慰剂维持治疗，结果发表时中位随访13.9个月，主要研究终点mOS：12.3个月*vs* 10.3个月，HR=0.70，*P*=0.006, 9；12个月的OS率两组分别为51.7% *vs* 38.2%，这是近20年来广泛期SCLC首个免疫联合疗法一线治疗取得总生存期获益的Ⅲ期研究，该方案由此写入2019年V1版美国国立综合癌症网络（National Comprehensive Cancer Network, NCCN）指南；同时我们应当注意这一研究存在的几点问题：首先，两组的客观缓解率（objective response rate, ORR）和中位缓解持续时间（median duration of response, mDoR）并没有显著差异，Atezolizumab的获益是体现在同步化疗阶段还是维持治疗阶段有待进一步的分析；其次，根据血浆肿瘤突变负荷（blood tumor mutation burden, bTMB）进行的亚组分析结果并没有看到明确的统计学差异，即没有找到免疫获益的优势人群；最后，本研究没有披露患者肿瘤标本PD-L1染色的情况以及临床获益是否和这一指标相关还需后续更新报道。

Duravelumab是另一种人源化抗PD-L1单克隆抗体，该药物开展的Ⅲ期、随机、开放、多中心CASPIAN研究旨在比较Durvalumab联合EP以及Durvalumab联合Tremelimumab再联合EP对比标准一线EP方案的疗效，Tremelimumab是一种新的抗CTLA-4单克隆抗体，这是SCLC领域第一个尝试CTLA-4抑制剂，PD-L1抑制剂再联合化疗的四药治疗模式，本研究的初步结果将在2019年公布。

### 2.2 维持治疗

CheckMate451是一项多中心、随机、Ⅲ期临床研究，预计纳入1, 327例广泛期SCLC经过一线化疗后肿瘤缩小或者稳定的患者，分别观察Nivolumab和Nivolumab联合Ipilimumab双药对比安慰剂巩固治疗的疗效，预计2019年公布初步结果。此外，有大量临床前研究证实放疗可通过产生新抗原、调节细胞因子释放、提高肿瘤对免疫细胞杀伤作用敏感性等方式调节机体抗肿瘤免疫应答，因此，放疗联合免疫治疗这一模式也在不断探索中，NCT03043599是观察在一线标准治疗后，Nivolumab联合Ipilimumab再联合胸部放疗维持治疗模式的Ⅰ期/Ⅱ期单臂研究，目前正在招募患者，预计2021年公布初步结果。

2018年*Journal of Thoracic Oncology*上发表了Pembrolizumab维持治疗的研究数据^[12]^，这一Ⅱ期多中心研究纳入了45例广泛期SCLC经过一线化疗后肿瘤缩小或者稳定的患者，给予固定剂量每3周1次的Pembrolizumab维持治疗，初步结果提示mPFS只有1.4个月，mOS为9.6个月，与既往的SCLC维持治疗研究相比并没有明显改善，但该研究患者的1年PFS率为13%，1年OS率为37%，mDoR为10.8个月，进一步亚组分析提示8例PD-L1表达≥1%的患者mPFS为6.5个月，mOS为12.8个月，这一结果提示我们，PD-L1表达情况似乎可以作为pembrolizumab维持治疗的标志物，但由于该研究样本量太小，还需要进一步的临床研究支持。

### 2.3 二线及以后治疗

CheckMate032是一项多队列、多中心、开放标签的Ⅰ期/Ⅱ期临床研究，评估Nivolumab联合Ipilimumab在经过一线或多线治疗后进展的SCLC患者疗效，2016年首次公布了非随机队列的研究结果^[13]^，患者非随机接受Nivolumab单药或Nivolumab联合Ipilimumab双药治疗，Nivolumab单药组3 mg/kg，1次/2周，双免疫组要接受剂量爬坡，药物起始剂量均为1 mg/kg+1 mg/kg，耐受后进入1 mg/kg+3 mg/kg或3 mg/kg+1 mg/kg模式；1次/3周，共4个周期；之后Nivolumab 3 mg/kg，1次/2周维持，汇总意向治疗人群的Nivolumab单药组ORR为11%，双免疫组为23%；2018年美国临床肿瘤学会（American Society of Clinical Oncology, ASCO）更新的2年OS数据显示，双免疫组2年生存率达到26%，Nivolumab组为14%；mDoR在Nivolumab单药组为17.9个月，双免疫组为14.2个月；在前期非随机队列取得了明确临床获益后，该研究进入扩展阶段，以3:2随机分配患者接受Nivolumab单药或Nivolumab 1 mg/kg联合Ipilimumab 3 mg/kg，双免疫组的ORR为21%，Nivolumab单药组为12%，与前期的非随机队列完全一致。基于该研究非随机队列长期随访数据，2018年8月17日，Nivolumab成为唯一被美国食品药品监督管理局（Food and Drug Administration, FDA）批准用于治疗既往接受过含铂方案化疗以及至少一种其他疗法后疾病进展的转移性SCLC患者；进一步汇总两部分研究^[14]^，其中211例患者可评估肿瘤突变负荷情况，应用全外显子测序确定其TMB数值，基于错义突变总数将患者分为低TMB（0-143）、中TMB（143-247）和高TMB（≥248）3个亚组，在高TMB亚组中，双免疫组ORR为46%，Nivolumab单药组为21%；中、低TMB组中，双免疫组ORR分别为16%和22%，Nivolumab单药组分别为7%和5%；双免疫组中高TMB的患者1年生存率为62%，Nivolumab单药组为35%，这一研究结果进一步证实TMB是预测SCLC免疫治疗效果的重要标志物，高TMB患者获得了显著的临床获益；该药的另一项Ⅲ期临床研究CheckMate331正在进行中，该研究旨在比较一线含铂化疗后进展的患者中Nivolumab对比单纯化疗药物Topotecan或者Amrubicin的疗效，主要研究终点为OS，预计2019年公布初步结果。

KEYNOTE-028是一项开放Ib期多队列研究，其SCLC队列纳入多次治疗后进展并且PD-L1表达阳性的广泛期患者^[15]^，研究招募165例，其中145例进行PD-L1表达筛查，46例表达≥1%，结合纳入与排除标准后最终只有24例接受Pembrolizumab治疗，2015年ASCO上汇报了该研究安全性和疗效的初步结果，ORR为37.5%，优于标准二线Topotecan的7%-24%，其mDoR达到了19.4个月，与化疗形成鲜明对比。KEYNOTE-158是涵盖11个瘤种的开放性、非随机、多中心多队列Ⅱ期研究，2018年ASCO上公布了SCLC队列的中期数据，该研究入组的107例患者对PD-L1表达无限制^[16]^，初步结果ORR为18.7%，mPFS为2.0个月，mOS为8.7个月，1年PFS和OS分别为16.8%和40.2%，mDoR没有达到，但73%的患者持续获益超过了12个月；在PD-L阳性的42例患者中，Pembrolizumab的ORR达到35.7%，mPFS和mOS分别为2.1个月和14.9个月。

Duravelumab在经治的广泛期SCLC患者中也开展了相关研究，并于2018年ASCO公布了初步的结果，这一多中心开放标签研究纳入21例多线治疗后进展的患者，该研究的ORR为9.5%，mPFS为1.5个月，mOS为4.8个月；虽然只有2例患者疾病得到了控制，但是其DOR分别为14.6个月和29.5个月。

## 3 总结与展望

SCLC恶性程度高，预后差，该领域相关治疗近20年没有新的突破，特别是二线及以后的治疗方案不理想，随着研究的不断深入，我们认识到SCLC具有一定的免疫源性、肿瘤突变负荷较高，免疫治疗有可能突破瓶颈，为SCLC患者带来曙光。已有的研究证实，免疫检查点抑制剂单药治疗的mDoR远远高于常规化疗，即免疫治疗一旦起效，患者长期获益率明显升高，因此，对优势人群的筛选即免疫标志物的探索迫在眉睫。

**1:** SCLC免疫检查点抑制剂临床研究数据总结 Summary of completed clinical trials of immune checkpoint inhibitors in SCLC

Study	Design	Sample size	Drugs	Patient population	Primary endpoint	Toxicity (Drug related)
CA184-041	Randomized double-blind multicenter phase 2	*n*=130 Sequential: 42 Concurrent: 43 Control: 45	Ipilimumab± Paclitaxel Carboplatin	Previously untreated ES-SCLC	Sequential *vs* Concurrent *vs* Control mPFS: 6.3 mo *vs* 5.7 mo *vs* 5.3 mo mOS: 12.5 mo *vs* 9.1 mo *vs* 10.5 mo	Sequential *vs* Concurrent *vs* Control Grade 3/4: 50% *vs* 43% *vs* 30%
CA184-156	Randomized placebo-controlled double-blind phase 3	*n*=1, 132 (Analyzed: 954) Combined: 478 Control: 476	Ipilimumab± Etoposide Platinum	Previously untreated ES-SCLC	Combined *vs* Control mPFS: 4.6 mo *vs* 4.4 mo mOS: 11 mo *vs* 10.9 mo	Combined *vs* Control Grade 3/4: 48% *vs* 44% Deaths: 1% *vs* 0.4%
IMpower133	Randomized double-blind placebo-controlled phase 3	*n*=403 Combined: 201 Control: 202	Atezolizumab± Carboplatin Etoposide	Previously untreated ES-SCLC	Combined *vs* Control mOS: 12.3 mo *vs* 10.3 mo mPFS: 5.2 mo *vs* 4.3 mo	Combined *vs* Control Grade 3/4: 39.9% *vs* 24.5% Death: 1.5% *vs* 1.5%
NCT02359019	Single arm open label multicenter phase 2	*n*=45	Pembrolizumab	Disease control after first-line chemotherapy ES-SCLC	mOS: 9.6 mo; mPFS: 1.4 mo 1-year PFS rate: 13% 1-year OS rate: 37%	Grade 3/4: 5% Deaths: 4.4%
CheckMate 032	Multicenter multi-cohort open label phase 1/2	Nonrandomized cohort: *n*=216; Nivo: 98; Nivo1+Ipi3: 61; Nivo3+Ipi1: 54 Randomized cohort: *n*=401; Nivo: 245; Nivo1+Ipi3: 156 TMB evaluable population: *n*=211; Nivo: 133; Nivo1+Ipi3: 78	Nivolumab± Ipilimumab	Progressed after platinum-based chemotherapy ES-SCLC or LS-SCLC	Nonrandomized: ORR: Nivo: 10%; Nivo1+Ipi3: 23%; Nivo3+Ipi1: 19% Randomized: (TMB) H *vs* I *vs* L ORR: Nivo: 21.3 % *vs* 6.8% *vs* 4.8% Nivo1+Ipi 3: 46.2% *vs* 16.0% *vs* 22.2% mPFS: Nivo: 1.4 mo *vs* 1.3 mo *vs* 1.3 mo Nivo1+Ipi3: 7.8 mo *vs* 1.3 mo *vs* 1.5 mo mOS: Nivo: 5.4 mo *vs* 3.9 mo *vs* 3.1 mo Nivo1+Ipi3: 22 mo *vs* 3.6 mo *vs* 3.4 mo 1-year OS rate: Nivo: 35.2% *vs* 22.1% *vs* 26.0% Nivo1+Ipi3: 62.4% *vs* 19.6% *vs* 23.4%	Nonrandomized: Grade 3/4: Nivo=13%; Nivo1+Ipi3=30%; Nivo3+Ipi1=19% Randomized: Grade 3/4: Nivo=14%; Nivo1+Ipi3=33%
KEYNOTE028	Open label multi-cohort phase Ib	*n*=24	Pembrolizumab	Progressed after platinum-based chemotherapy ES-SCLC PD-L1: ≥1%	ORR: 37.5% mPFS: 1.9 mo mOS: 9.7 mo mDoR: 9.0 mo	All grades: 66.7%
KEYNOTE158	Open label multi-cohort multicenter phase 2	*n*=107	Pembrolizumab	Progressed after platinum-based chemotherapy ES-SCLC	ORR: 18.7% mPFS: 2.0 mo mOS: 8.7 mo 73% DoR≥12 mo	All grades: 59%
SCLC: small cell lung cancer; mPFS: mean progression-free-survival; mOS: mean overall survival; mDoR: median duration of response; ES-SCLC: extensive stage SCLC; LS-SCLC: limited-stage SCLC; PD-L1: programmed death-ligand 1.						

CheckMate032研究提示SCLC患者中PD-L1的表达并不常见，只有18%的患者PD-L1≥1%，进一步分析提示其表达水平与临床获益不相关：PD-L1 < 1%和PD-L1≥1%的患者单药组ORR为14% *vs* 9%，双免疫组为32% *vs* 10%，即PD-L1 < 1%的患者ORR均高于PD-L1≥1%的患者；而KEYNOTE-158研究却得到了相反的结果，在入组的107例患者中，PD-L1≥1%的占39%，并且临床疾控率与PD-L1的表达明显相关。诚然，这两个研究用到的抗体不同，研究规模不同，不能直接进行比较，但正因为存在这种显著差异，目前，PD-L1对于SCLC来说并不能作为一个理想的标志物，其获益只限于Pembrolizumab的小样本研究。

另一方面，CheckMate032研究指出TMB与SCLC免疫疗效有明显相关性，TMB高的患者接受双免疫联合治疗效果显著，但目前对于TMB的评价标准并不统一，该研究是对患者组织标本进行的全外显子检测，因此，如果应用TMB进行筛选，应严格遵循这一原则；对于无法获取足够肿瘤组织进行分子检测的患者，bTMB就显得极为重要。Gandara等^[17]^指出，bTMB与组织TMB的阳性一致率为64%，阴性一致率为88%，这一结论源于Atezolizumab在NSCLC领域中的OAK研究^[18]^和POPLAR研究^[19]^数据归纳总结；此外，bTMB在不同Cut-Off值下（≥10、≥16和≥20）区分的PFS或OS，对于高表达的人群均能从Atezolizumab中获益。但在IMpower133研究的亚组分析中，bTMB并没有区分出真正的获益人群，这是否与其联合化疗的模式有关还需进一步研究证实。

总之，目前的免疫标志物PD-L1以及TMB表达有各自的应用局限性，并不能精准地筛选出免疫获益人群，我们需要找到更全面、更准确、预测效力更强的指标筛选出真正的免疫获益群体，才能将免疫检查点抑制剂在SCLC领域的抗肿瘤作用发挥到最佳。

## References

[1] Semenova EA, Nagel R, Berns A (2015). Origins, genetic landscape, and emerging therapies of small cell lung cancer. Genes Dev.

[2] Kalemkerian GP, Schneider BJ (2017). Advances in small cell lung cancer. Hematol Oncol Clin North Am.

[3] Govindan R, Ding L, Griffith M (2012). Genomic landscape of non-small cell lung cancer in smokers and never-smokers. Cell.

[4] Bunn PJ, Minna JD, Augustyn A (2016). Small Cell lung cancer: can recent advances in biology and molecular biology be translated into improved outcomes?. J Thorac Oncol.

[5] Gazdar AF, Bunn PA, Minna JD (2017). Small-cell lung cancer: what we know, what we need to know and the path forward. Nat Rev Cancer.

[6] List M, Jamous F, Gupta A (2015). Anti-Hu positive antibodies and small cell carcinoma: a single center review. S D Med.

[7] Graus F, Dalmou J, Rene R (1997). Anti-Hu antibodies in patients with small-cell lung cancer: association with complete response to therapy and improved survival. J Clin Oncol.

[8] Doyle A, Martin W J, Funa K (1985). Markedly decreased expression of class Ⅰ histocompatibility antigens, protein, and mRNA in human small-cell lung cancer. J Exp Med.

[9] Reck M, Bondarenko I, Luft A (2013). Ipilimumab in combination with paclitaxel and carboplatin as first-line therapy in extensive-disease-small-cell lung cancer: results from a randomized, double-blind, multicenter phase 2 trial. Ann Oncol.

[10] Reck M, Luft A, Szczesna A (2016). Phase Ⅲ randomized trial of ipilimumab plus etoposide and platinum versus placebo plus etoposide and platinum in extensive-stage small-cell lung cancer. J Clin Oncol.

[11] Horn L, Mansfield AS, Szczesna A (2018). First-line atezolizumab plus chemotherapy in extensive-stage small-cell lung cancer. N Engl J Med.

[12] Gadgeel SM, Pennell NA, Fidler MJ (2018). Phase Ⅱ study of maintenance pembrolizumab in patients with extensive-stage small cell lung cancer (SCLC). J Thorac Oncol.

[13] Antonia SJ, Lopez-Martin JA, Bendell J (2016). Nivolumab alone and nivolumab plus ipilimumab in recurrent small-cell lung cancer (CheckMate 032): a multicentre, open-label, phase 1/2 trial. Lancet Oncol.

[14] Hellmann MD, Callahan MK, Awad MM (2018). Tumor mutational burden and efficacy of nivolumab monotherapy and in combination with ipilimumab in small-cell lung cancer. Cancer Cell.

[15] Ott PA, Elez E, Hiret S (2017). Pembrolizumab in patients with extensive-stage small-cell lung cancer: results from the phase Ib KEYNOTE-028 study. J Clin Oncol.

[16] Diaz L, Marabelle A, Kim TW (2017). Efficacy of pembrolizumab in phase 2 KEYNOTE-164 and KEYNOTE-158 studies of microsatellite instability high cancers. Ann Oncol.

[17] Gandara DR, Paul SM, Kowanetz M (2018). Blood-based tumor mutational burden as a predictor of clinical benefit in non-small-cell lung cancer patients treated with atezolizumab. Nat Med.

[18] Rittmeyer A, Barlesi F, Waterkamp D (2017). Atezolizumab versus docetaxel in patients with previously treated non-small-cell lung cancer (OAK): a phase 3, open-label, multicentre randomised controlled trial. Lancet.

[19] Fehrenbacher L, Spira A, Ballinger M (2016). Atezolizumab versus docetaxel for patients with previously treated non-small-cell lung cancer (POPLAR): a multicentre, open-label, phase 2 randomised controlled trial. Lancet.

